# Heterogeneity of national legislation and practice on clinical trials with vulnerable patients based on the EU Clinical Trials Directive by the example of adults permanently incapable of giving informed consent

**DOI:** 10.3205/000290

**Published:** 2021-03-02

**Authors:** Janna K. Schweim, Michael Nonnemacher, Karl-Heinz Jöckel

**Affiliations:** 1 Institute for Medical Informatics, Biometry and Epidemiology (IMIBE), University Hospital Essen, University Medicine Essen, Germany

**Keywords:** clinical trial, informed consent, vulnerable person, patient incapable of giving informed consent, legal representative

## Abstract

In principle, persons wishing to participate in a clinical trial must give informed consent in advance after comprehensive information has been provided. Under certain conditions, it is possible to deviate from this requirement in the European Union (EU) in order to enable the participation of so-called vulnerable persons who are incapable of giving their informed consent. Kuthning et al. [1] have already dealt with general and specific aspects of vulnerable patients and the principle of informed consent in clinical trials. One group of vulnerable persons, for example, are adults temporarily or permanently incapable of giving consent due to their state of health. For a long period of time, no systematic and uniform legal basis for clinical trials existed in the EU as a whole. The Clinical Trials Directive (CTD) [2], adopted in 2001, aimed to change this by harmonizing all legal regulations on clinical trials applicable in the EU, but nevertheless allowing national deviations in implementation into national laws through opening clauses and aspects that were left unregulated. In view of the Clinical Trials Regulation (CTR) [3] which, according to the current status, will with high probability be applied from 2022 on, and which in future will be the legal basis for clinical trials with medicinal products in humans, applied directly in all EU member states, the necessity to take stock of the effects of the CTD was evident.

The national deviations with regard to the participation of patients incapable of giving informed consent were investigated qualitatively and quantitatively by means of a systematic analysis of legislation in 16 EU countries and a retrospective database analysis of a European clinical trial registry over a ten-year observation period. Although the analysis initially showed a predominantly homogeneous picture, the differences between the EU member states became apparent in a detailed examination. The database analysis yielded a clear result, since in some countries the majority of clinical trials are carried out. The clearest difference was found between the legal analysis and the results of the evaluated clinical trials concerning adults who are permanently incapable of giving informed consent. A presumed association between the “degree of liberality” of the national law and the frequency of clinical trials conducted in the respective country could not be confirmed. In the past, the selection of countries for conducting a clinical trial was based less on legal requirements and more on experience and financial considerations.

## Zusammenfassung

Grundsätzlich müssen Personen, die an einer klinischen Prüfung teilnehmen wollen, vorab eine informierte Einwilligung nach umfassender Aufklärung erteilen. Unter bestimmten Voraussetzungen kann in der Europäischen Union (EU) von diesem Erfordernis abgewichen werden, um sogenannten vulnerablen Personen, die nichteinwilligungsfähig sind, die Prüfungsteilnahme zu ermöglichen. Mit generellen und spezifischen Aspekten schutzbedürftiger Patienten sowie dem Grundsatz der Einwilligung nach Aufklärung in klinischen Prüfungen haben sich bereits Kuthning et al. [1] auseinandergesetzt. Eine Gruppe vulnerabler Personen sind zum Beispiel Erwachsene, die aufgrund ihres Gesundheitszustands entweder vorübergehend oder dauerhaft nichteinwilligungsfähig sind. Über einen langen Zeitraum existierten in der EU insgesamt keine systematischen und einheitlichen Rechtsgrundlagen für klinische Prüfungen. Dies sollte durch die 2001 erlassene Clinical Trials Directive (CTD) [2] geändert werden, welche trotz geplanter Harmonisierung aller in der EU geltenden gesetzlichen Vorschriften zu klinischen Prüfungen, durch Öffnungsklauseln sowie ungeregelt belassene Aspekte, nationale Abweichungen bei der Implementierung in die Landesgesetze ermöglichte. Im Hinblick auf die, nach derzeitigem Stand, mit hoher Wahrscheinlichkeit ab 2022 wirksame Clinical Trials Regulation (CTR) [3], welche zukünftig als rechtliche Grundlage für klinische Prüfungen mit Arzneimitteln am Menschen unmittelbar in allen EU-Mitgliedsstaaten gelten soll, lag es nahe, eine Bestandsaufnahme der Auswirkungen der CTD durchzuführen.

Die nationalen Abweichungen hinsichtlich der Teilnahme nichteinwilligungsfähiger Personen wurden durch eine systematische Gesetzesanalyse in 16 EU-Staaten sowie eine sich über einen Beobachtungszeitraum von zehn Jahren erstreckende, retrospektive Datenbankauswertung eines europäischen klinischen Studienregisters qualitativ und quantitativ untersucht. Obwohl sich anhand der Analyse zunächst ein überwiegend homogenes Bild zeigte, traten die Unterschiede zwischen den EU-Staaten bei detaillierterer Betrachtung zum Vorschein. Die Datenbankauswertung ergab ein eindeutiges Ergebnis, da in einigen Ländern der Großteil klinischer Prüfungen durchgeführt wird. Der deutlichste Unterschied trat zwischen der Gesetzesanalyse und den Ergebnissen der ausgewerteten klinischen Prüfungen bezüglich dauerhaft nichteinwilligungsfähiger Erwachsener zutage. Ein erwarteter Zusammenhang zwischen dem „Liberalitätsgrad“ des nationalen Gesetzes und der Häufigkeit der in dem jeweiligen Land durchgeführten klinischen Prüfungen konnte nicht bestätigt werden. Die Auswahl der Länder als Durchführungsort für eine klinische Prüfung orientierte sich in der Vergangenheit weniger an den gesetzlichen Voraussetzungen als vielmehr an Erfahrungswerten und finanziellen Erwägungen.

## Introduction

A justifiable consent of the person concerned is required as permission to carry out clinical trials with medicinal products on humans. This so-called informed consent is an expression of the constitutionally guaranteed right of self-determination and the right to physical integrity. Under German criminal law, any unjustified intervention that is likely to affect the body or health of a person constitutes a punishable bodily injury. Therefore, without an effective, justifying consent to participation in a clinical trial, the medical intervention of the performing physician, i.e. investigator, would always be regarded as a punishable interference with the physical integrity. The subject should give informed consent based on a comprehensive explanation of the objectives, methods, expected benefits and potential risks of the intended treatment and possible alternatives. In this respect, informed consent on the one hand serves as protection of the investigator under criminal law, and on the other hand enables the patient to decide for or against participation after weighing all advantages and disadvantages.

The patients for whose needs medicinal products are to be developed also include those who cannot give informed consent themselves for participation in the clinical trial. This inability to give informed consent may occur due to age or state of health: Minors have not yet reached the necessary mental maturity and capacity for understanding to perform such an important decision on their own. Adults, i.e. persons of legal age, may be permanently incapacitated as a result of an illness affecting them, or temporarily incapacitated due to an acute event or emergency situation. The members of these patient groups are referred to as vulnerable persons, who are, according to the Declaration of Helsinki (DoH) [4], described as particularly in need of protection. Kuthning et al. [1] have dealt with general and specific aspects of vulnerable patients and the principle of informed consent in clinical trials. The DoH presupposes that it is essential to include vulnerable persons in medical research insofar as it concerns them, is of direct benefit to them and cannot be carried out on non-vulnerable individuals. The Council of International Organizations of Medical Sciences’ (CIOMS) Code of Ethics [5] deals in more detail with research involving vulnerable individuals and groups, and specifically includes those who have limited or no capacity to give consent to participate in research. The guideline goes into more detail on adults who are unable to give consent as well as on children and adolescents as research participants. In adults, the inability to give informed consent is not accepted until clear evidence of impaired ability to give consent is found, e.g. due to dementia, psychiatric illness, accidents or sudden emergency situations such as sepsis, craniocerebral trauma, cardiopulmonary arrest or stroke. Clinical trials should always be conducted under participation of those patients who represent the target population. Both minors and adults who are permanently incapacitated need a legal representative to decide on these patients’ behalf about their participation in the trial.

In the European Union (EU), the legal framework for the conduction of clinical trials with medicinal products has been set by the EU Clinical Trials Directive 2001/20/EC (CTD) [2] since April 4, 2001. Previously, each country dealt with the subject matter itself to a greater or lesser extent, and the existing national regulations differed considerably because no uniform and systematic legal basis existed in the EU. Therefore, the CTD aimed at harmonizing the requirements for clinical trials in all EU member states and simplifying clinical trials conducted across borders: In particular, it was intended to promote research and development of medicinal products in the EU and to ensure protection and integrity of human beings. Regarding adults incapable of giving informed consent, recital 4 of the CTD states: “In the case of other persons incapable of giving their consent, such as persons with dementia, psychiatric patients, etc., inclusion in clinical trials in such cases should be on an even more restrictive basis. Medicinal products for trial may be administered to all such individuals only when there are grounds for assuming that the direct benefit to the patient outweighs the risks” [2]. In order to achieve these objectives, according to Lemaire et al. [6], a correspondingly complete implementation of the CTD in the member states was presupposed, including the repeal of conflicting and unnecessary national regulations. The CTD was implemented into national law in the existing member states on May 1, 2004, with adapted deadlines applying to the EU member states that joined within the framework of the EU enlargement. However, the CTD allowed the member states leeway for implementation in some points in order to secure locational advantages.

Due to the “opening clauses” of the CTD, it is assumed that the laws of the EU member states continued to contain different conditions which in some cases allow a partly simplified, in other cases a partly more difficult conduction of clinical trials, especially regarding the participation of vulnerable persons. The specification of these requirements is also likely to result in a number of EU states in which clinical trials with patients incapable of giving informed consent themselves will be conducted more frequently. Furthermore, a consultation process on the advantages and disadvantages of the CTD, which led to the decision to adopt the CTR in 2009, showed as a central point of criticism the decline in the number of clinical trials in the EU by 25% from 2007 to 2011 [7]. Bureaucratic hurdles for multinational clinical trials within the EU, such as inconsistent legal and regulatory requirements, were identified as the reason for progressing relocation to countries outside Europe with lower requirements [8]. The research idea to investigate the differences in national laws and their impact on the number of clinical trials in more detail arose from this initial situation. Based on these facts, the hypothesis was created that countries with a more liberal legislation for the conduction of clinical trials with persons incapable of giving informed consent conduct clinical trials involving these patients more frequently. In turn, countries with stricter laws would be conducting fewer clinical trials, or none at all, including these vulnerable patient groups. This hypothesis was tested using in particular the example of adults who are permanently incapable of giving informed consent [9]. The main research question addresses whether the expression of legal regulations in a vulnerable patient population affects the number of clinical trials in this population.

## Methods

The implementation of the CTD in national legislation was examined by means of an exemplary selection of 16 EU member states; the relevant national legal texts have been evaluated retrospectively and systematically [10]. The EU states were selected in such a way that on the one hand, an analysis of the law was possible on the basis of the authors’ own understanding of the languages or the availability of an English translation and, on the other hand, a representative cross-section of the EU could be used for the examination. In order to obtain the broadest possible picture as a cross-section of the EU, Germany, France, Italy, Portugal and Spain were selected as the five founding members of the consortium European Clinical Research Infrastructure Network (ECRIN). The Czech Republic, Hungary, Ireland and Poland were selected as further countries, and the selection was completed with Austria, Belgium, the Netherlands, Denmark, Finland, Sweden and the United Kingdom, before the latter left the EU as a result of Brexit. The legal texts of these 16 countries which are concerned with the conduct of clinical trials were each obtained in the version by which the CTD was actually transposed and applied into national law. The analysis of the legal texts focused on general regulations for the inclusion of trial subjects as well as specific regulations for the conduct of clinical trials involving vulnerable patients. In order to enable a quantitative evaluation of the national legal texts with regard to liberality or restrictiveness, three suitable variables (so-called items) were selected to be evaluated and summarized according to a scoring system. The items were scored by analogy with clinical scales, such as the CLIP score for assessing the survival probability of patients with hepatocellular carcinoma according to the Cancer of the Liver Italian Program (CLIP score) as described by Llovet and Bruix [11]. Depending on whether and how an item was fulfilled in the respective member state, a score of 0, 1 or 2 was awarded, whereby – in contrast to the CLIP score – a higher value has been assumed as desirable for the legal items.

In addition, a database search was carried out in the clinical study register EU Clinical Trials Register (EU-CTR) for clinical trials in the previously selected EU countries. A ten-year period from January 1, 2008 to December 31, 2017 was chosen as the period to be investigated retrospectively. The group of adults permanently incapable of giving informed consent was represented by the search for clinical trials phase III with persons ≥65 years of age with dementia caused by Alzheimer’s disease, indication “dementia in Alzheimer’s disease with late onset” (type 1) according to the definition F.00.1 of the International Statistical Classification of Diseases and Related Health Problems ICD-10 (search terms: “elderly”, “dementia AND Alzheimer”). A relevant set of variables was extracted from the information documented in the database. These variables have been evaluated as if each clinical trial was representing a Case Report Form (CRF). The variables consisted preferably of numerical data or data documented according to a pre-selection from a drop-down menu such as answers “yes” or “no”. Apart from the database sections “A. Protocol Information” and “B. Sponsor Information”, the most relevant variables have been extracted from the sections “E. General Information on the Trial”, e.g. “principle inclusion/exclusion criteria”, “trial being conducted within and outside the EEA”, and “F. Population of Trial Subjects”, e.g. “age range” or “specific vulnerable populations”. The collected data were described by descriptive analysis and statistically evaluated in the form of frequency counts.

## Results

### Score of the national laws on the basis of defined items for adults incapable of giving informed consent

The results of the legal text analysis of the individual EU member states have been combined for a direct comparison. Three characterizing items from the legal texts have been identified as variables for the evaluability of the results on patients incapable of giving informed consent. These items were suitable for providing an indication of whether the legal norms in the member states allow conclusions to be drawn about the liberality of the countries with regard to the conduct of clinical trials.

In detail, item A refers to persons/institutions with the authority to represent the adult incapable of giving informed consent: While the legal representative – designated by court after determination of the suitability of the respective representative – is the usual, but most strict legal prerequisite (=0), physician, authority and court are standing on the next level equally, since these persons/institutions make the decision either with medical expertise or based on their state authority (=1). The authority of representation of the relative/spouse, which is given at any time without any need to prove a special qualification, is rated with the highest degree of liberality (=2).

For item B, the refusal for participation declared in the state of incapacity, the specific inquiry of the person unable to give consent to take part in the examination is the strictest option (=0). The next higher level is the existence of the subject’s own declaration which was expressed during a state of consent (=1). The most liberal option is the case when a declaration by the relatives regarding the presumed will of the person concerned is considered sufficient (=2).

Regarding the group benefit (item C), which is not regulated at EU level for adults incapable of giving informed consent, the most liberal option is “explicitly allowed” (yes=2), and the next lower level is “explicitly prohibited” (no=1). National laws without a statement about group benefit must be associated with legal uncertainty, as a different classification could be made from study to study. From the patient’s point of view, the missing legal regulation is the worst conceivable option and at the same time the most restrictive (=0).

The total score of a member state resulting from the addition of items A, B and C was formed for the patient group, which resulted in a hierarchy of the examined EU member states (Table 1 (Tab. 1)). 6 was the highest possible score and 0 the lowest. The higher the value, the more liberal the national regulations appear. A lower value thus suggests a more restrictive law, and at the same time indicates that the conduct of clinical trials with vulnerable patient populations is, if at all, only possible under more difficult conditions.

**Figure 1:**
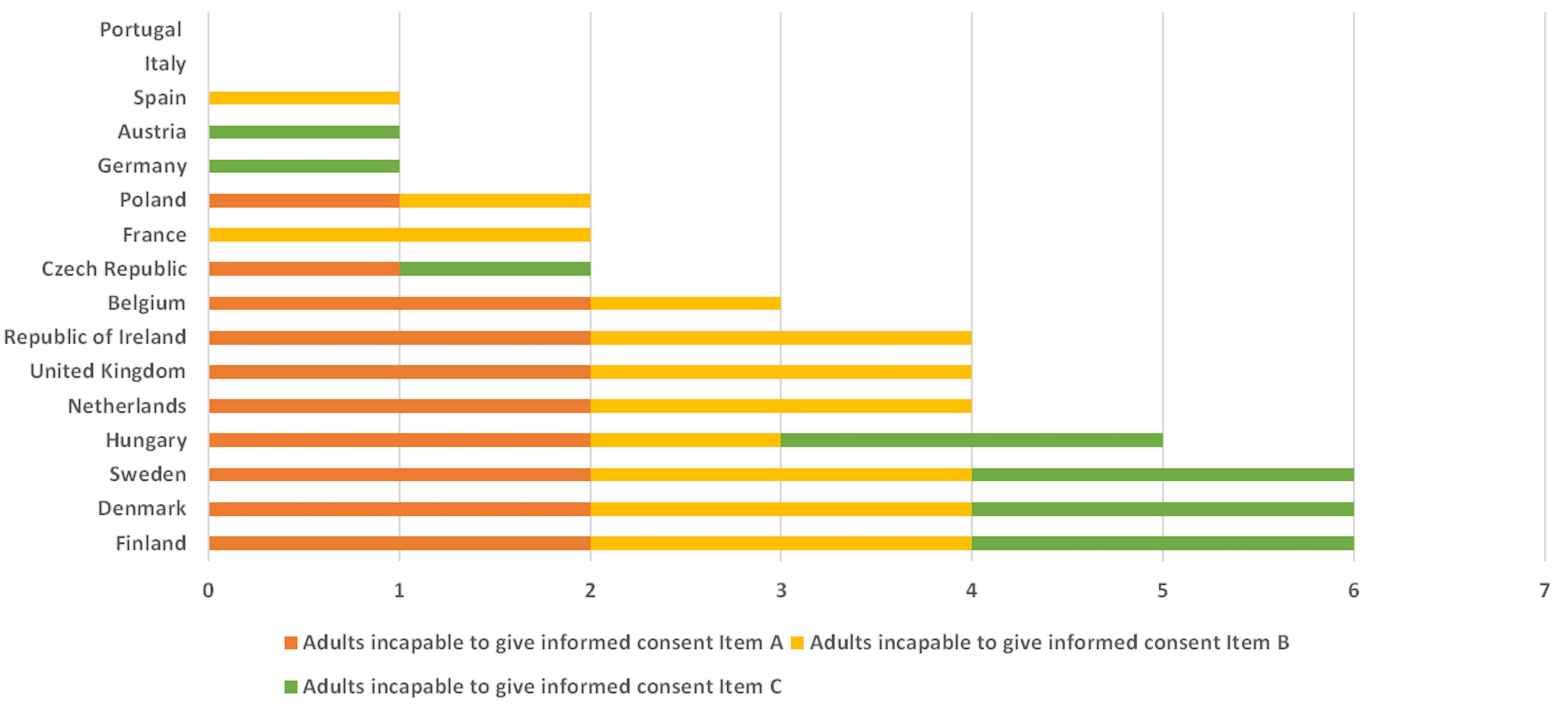
Comparison of the legal regulations in selected EU member states on the basis of the items on adult patients permanently incapable of giving informed consent

In the overall view of all EU member states examined (Figure 1 (Fig. 1)), the Scandinavian countries Finland, Denmark and Sweden, followed by Hungary, seem to have the most liberal legislation regarding the participation of adults who are permanently incapable of giving informed consent. Belgium and the Netherlands, the United Kingdom and Ireland, as well as the Czech Republic, France and Poland are in the middle of the field, while Germany and Austria and the southern European countries Spain, Italy and Portugal are in the lower ranks. Although countries such as Italy and Portugal also have legislations in place for the representation of adults permanently incapable of giving informed consent, these countries have the weakest comparative impact on the feasibility of clinical trials.

### Number of clinical trials with elderly patients suffering from Alzheimer’s dementia in the EU-CTR Clinical Trials Registry

Searches for elderly people over 65 years of age with Alzheimer’s disease found 93 clinical trials for the period January 1, 2008 to December 31, 2017. For 46 of these trials, the EU-CTR database contained a result report at the time of the search. Of these 93 clinical trials, 24 were purely national, the remaining 69 were multinational. In addition, 13 clinical trials have apparently been first registered in the database before the inclusion period for the search, i.e. before January 1, 2008. The initial data entry into the database for the specific clinical trial often took place later, whereby the search function of the EU-CTR database does not consider the time lag between the application for the registration number and the actual registration. Clinical trials for which the application for a registration number had been made before 2008 were excluded from the analysis; thus, N=80 clinical trials on elderly patients with Alzheimer’s dementia were analyzed in more detail.

Of these 80 clinical trials remaining after adjustment, 21 were purely national, conducted in only one EU member state, and 59 were multinational. However, this result must also be considered with the restriction that, although some clinical trials had only received approval in one EU member state at the time of the search, in several cases they were planned to be conducted in other states and outside the EU or European Economic Area (EEA).

**Figure 2:**
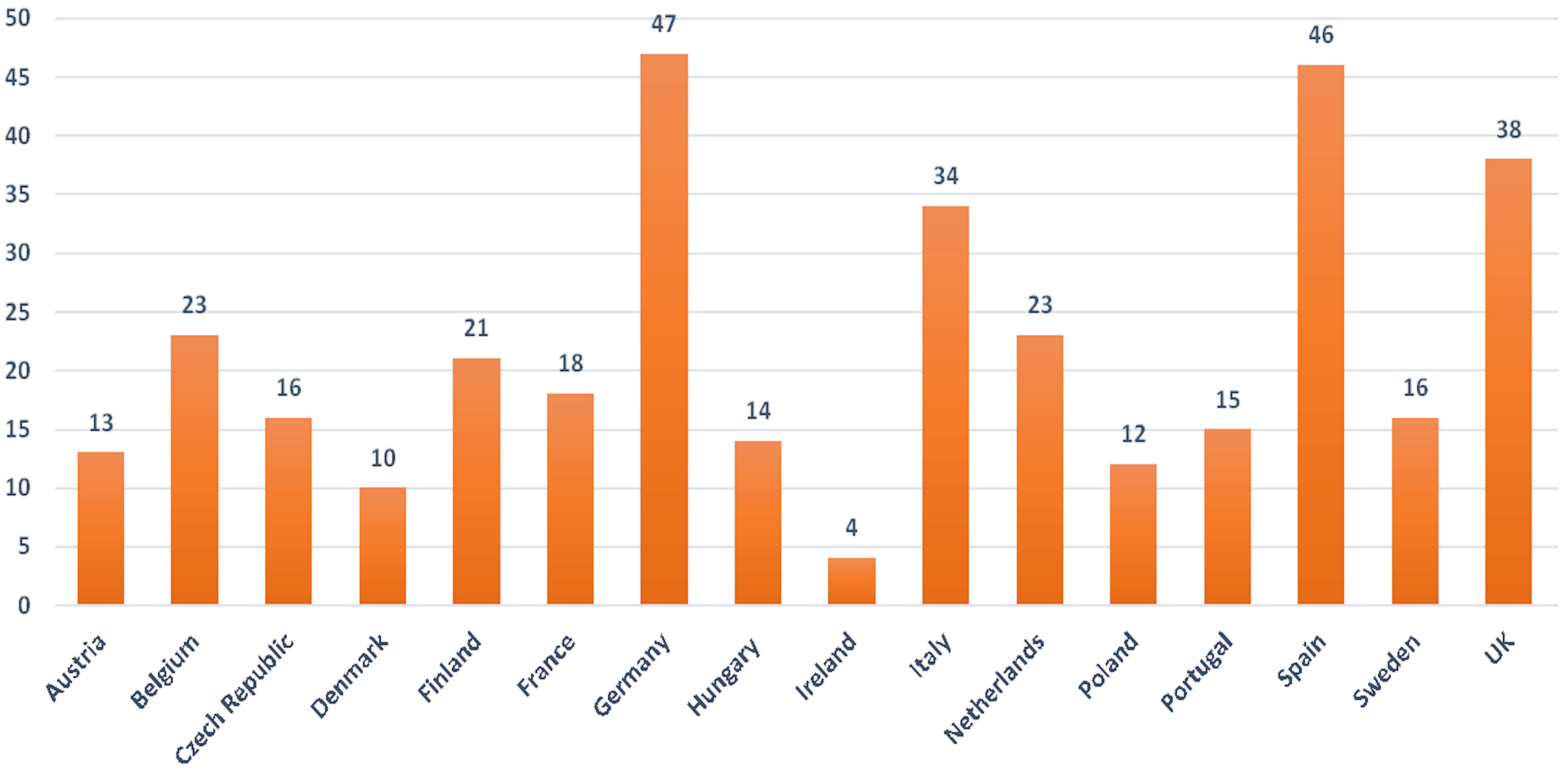
Approved clinical trials in EU-CTR in selected EU countries with adults with Alzheimer’s dementia incapable of giving informed consent (2008–2017)

**Figure 3:**
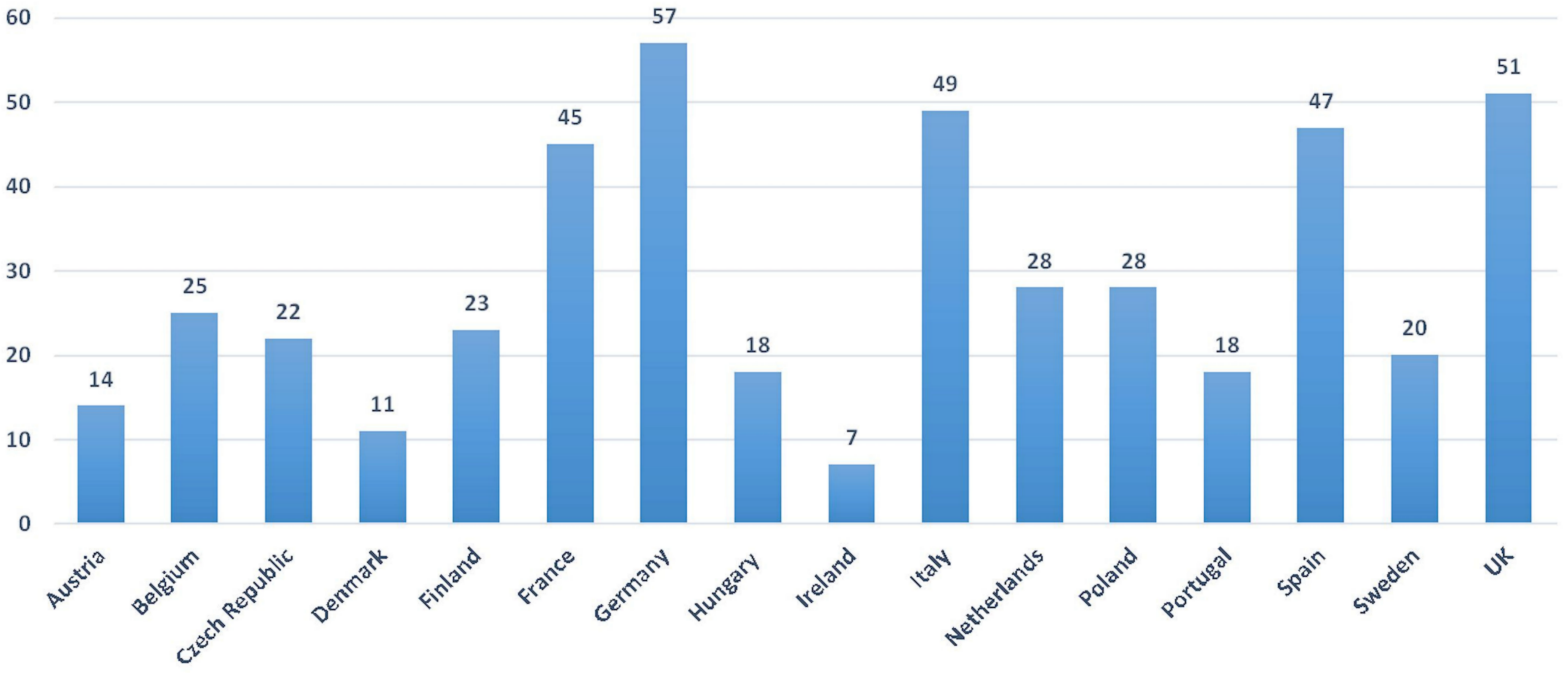
Total of approved and planned clinical trials in EU-CTR in selected EU countries with adults with Alzheimer’s dementia incapable of giving informed consent (2008–2017)

The evaluation of the clinical trials with adults permanently incapable of giving informed consent that had already been approved in the selected 16 EU member states at the time of the search (Figure 2 (Fig. 2)) were compared with the numbers that have changed as a result of the additional consideration of planned applications (Figure 3 (Fig. 3)).

In clinical trials with adults permanently incapable of giving informed consent, Germany is in first place among the approving EU countries (47); the gap to Spain is quite small (46), followed by the United Kingdom (38) and Italy (34). A number of EU states are in the middle field, Denmark (10) and Ireland (4) are in the lower places. The differences between the frequently and rarely requested countries are very obvious.

By comparing the numbers in Figure 2 (Fig. 2) with those in Figure 3 (Fig. 3), it becomes clear that – despite a few exceptions after addition of the planned clinical trial applications in the 16 selected EU countries – the hierarchy of the EU states, which indicates the frequency with which countries are required to conduct a clinical trial, remains unchanged.

In the evaluated clinical trials with Alzheimer’s dementia patients incapable of giving informed consent, several discrepancies change the hierarchy of the EU states more strongly due to the number of planned approvals. The gap between Germany and the other EU states has increased. The order of the countries has changed from second place onwards: While Germany continues to occupy the first place among clinical trial approvals (57), the United Kingdom (51) moves up to second place and Italy (49) to third. Spain (47) is relegated to fourth place, followed by France (45) in fifth place. Poland (28), the Netherlands (28) and Belgium (25) are in the middle of the field, while the lower positions remain unchanged.

### Main results

A direct comparison of the results of the legal analyses with those of the database evaluation reveals considerable discrepancies. With regard to the legislation on incapacitated adults, there was no congruence with the results of the database analysis. In none of the countries classified as legally liberal (Figure 1 (Fig. 1)) did a majority of clinical trials take place in this population (Figure 2 (Fig. 2), Figure 3 (Fig. 3)). Thus the original hypothesis, which assumed a relationship between legislative liberalization and the number of clinical trials conducted, could not be confirmed. This negative result is discussed and interpreted below.

## Discussion

Considering the different options of item A that were developed for the comparison of the national laws, a legal representative should give consent on the patient’s behalf about that patient’s participation in a clinical trial when the patient is incapable of giving informed consent. Persons of legal age who are permanently incapable of giving informed consent need someone who can give consent to participation in the trial on their behalf. In all EU member states studied, a legal representative appointed by a court should primarily assume this role, although the law also stipulates additional solutions. However, eight countries allow a person of trust with a close relationship, usually a family member, to give consent (France, United Kingdom, Ireland, Denmark, Finland, Spain, Belgium, the Netherlands), (Table 1 (Tab. 1)). As assumed, this will often be a solution in cases where no legal representative has (yet) been appointed. The extension of the circle of persons authorized to give informed consent is partly a relief, but this is also accompanied by a high level of responsibility for the persons entitled, especially close relatives. The difference between a court-appointed guardian, who may also be a relative, and a person with a close relationship, is that the former has been assessed with regard to his or her qualification for this task and has been classified as suitable. A family relationship may also cause difficulties for the relative to decide objectively on certain medically necessary measures due to the personal relationship.

Item B is not discussed in detail in this paper. However, it was found that the most liberal option – as stated by the CTD – that the presumed will of the adult permanently incapable of giving informed consent should be considered, has not been implemented by most of the national laws being investigated. It is even more difficult to get hold of an earlier, explicit statement by the person concerned for investigating the willingness of an incapacitated person to participate in a clinical trial, than accepting the declaration by the relatives regarding the presumed will of the person concerned.

If conducting a clinical trial is not only permissible in cases where the trial subject can expect an advantage for himself or herself, but research may be conducted for the benefit of a group of similarly ill patients, this opens up greater freedom in research. The additional objective of achieving a benefit for the group of similarly ill patients – as examined by item C – corresponds to the goal of using a clinical trial to obtain results that are transferable to other patients beyond the individual case. For adults permanently incapable of giving informed consent, the CTD has not explicitly addressed the option of a group benefit. Only four countries have established a national regulation on group benefit for adults incapable of giving informed consent (Denmark, Finland, Sweden and Hungary), which shows that for this group of patients, such a regulation is considered rather sensitive. It would be a serious problem if the subjects were no longer expected to benefit themselves at all, but to the contrary, if participation were to be used solely to benefit other patients. Such an approach would degrade the trial subject to a mere object of the medical experiment and instrumentalize him or her for research purposes. The interpretation is possible that some countries have decided to waive group benefit regulations because of this risk. In order to avoid a violation of the basic right of human dignity as standardized e.g. in Art. 1 (1) of the German Constitutional Law, the benefit for the individual should always outweigh the risks of participation in the trial, even when compared to a benefit for society. If in addition to this, there is a benefit for the general public, this will promote progress in scientific research.

The database analysis of clinical trials in the selected EU member states has shown that some countries are represented particularly frequently, while others are represented particularly rarely. Most of the clinical trials involving adults permanently incapable of giving informed consent were applied for in Germany, and countries such as Spain, Italy, France and the United Kingdom were also very often represented. In contrast, EU countries such as Austria, Ireland and Portugal often ended up in the bottom ranks. Various explanations are possible why some countries have been particularly frequented for conducting clinical trials and others have been virtually neglected. With regard to the legislation on adults permanently incapable of giving informed consent, there was no congruence with the results of the database analysis: In none of the countries classified as legally liberal (Figure 1 (Fig. 1)) did a majority of clinical trials take place in this population (Figure 2 (Fig. 2), Figure 3 (Fig. 3)), so indeed a considerable deficit could be found here.

Arguments in favor of a recurring election of the same countries would initially be traditional habits or good experiences with the authorities or Ethics Committees (EC), which might have led to a routine in the application process. In addition, long-standing good relationships and existing contacts of the sponsors with certain medical institutions can lead to clinical trials being conducted repeatedly in the same country. In the area of commercial clinical trials in particular, economic reasons play an important role. These may include the amount of the application fees or the duration of the approval process, as the rapid processing allows an earlier start of the clinical trial. The costs of the respective health care system in terms of “case payments” or the value added tax rate are also among the economic factors that may result in some EU member states appearing more attractive than others.

A possible explanation why other member states are less frequently chosen for conducting clinical trials could be language barriers. Some national languages are among the less common, and since in some cases, English may not yet be accepted as the universal language by all authorities, the information exchange during the application process will be more difficult. Again, the cost factor must be considered, especially with regard to the translation of documents necessary for the information and involvement of the participants into the national languages. There may not yet be such good contacts for cooperation with some authorities and institutions of EU member states that, for example, joined the EU later. Also, the smaller size of some countries could be a reason to exclude them from a clinical trial, due to the smaller number of potential trial participants.

Overall, financial reasons and experience, rather than a systematic analysis of laws, seem to be the most important factors in the selection of the countries conducting clinical trials. Considering the analysis of country legislation, it might be worthwhile for sponsors planning clinical trials with adults permanently incapable of giving informed consent to pay more attention to the Scandinavian countries Finland, Denmark and Sweden due to their more liberal laws, as well as to Hungary.

## Conclusions

The development of age-appropriate medications for the treatment of characteristic age-related diseases (e.g. Alzheimer’s) is becoming increasingly important in the light of demographic change. As already discussed, a distinction is made in adults between those who – due to health conditions – have permanently, and those who – due to an acute illness – have temporarily lost the ability to understand all the circumstances of the situation they find themselves in, to form an opinion and give informed consent. In both cases, a legal representative should be entitled to give consent for participation in a clinical trial on behalf of the incapacitated person.

In the past, the abandonment of research on patients incapable of giving informed consent was often motivated by the well-meant intention of protecting these patients from harm. However, if research involving these patients were to be abandoned, the result would be a conscious decision to forego progress in the detection and treatment of specific diseases. The intended protection would thus actually have a negative impact on the chances of curing those who are incapable of giving informed consent. In contrast, a well-planned clinical trial would offer the chance that the burdensome measures could be outweighed or even overweighed by the benefits to be achieved. It is therefore necessary in the future to continue creating and exploiting legal possibilities for the conduction of medical research involving patients who are incapable of giving informed consent. Further research would also be valuable to determine whether and how the application of the Clinical Trials Regulation (CTR) [3] might change and potentially improve the participation in clinical trials for vulnerable patient groups.

## Notes

### Competing interests

The authors declare that they have no competing interests.

## Figures and Tables

**Table 1:**
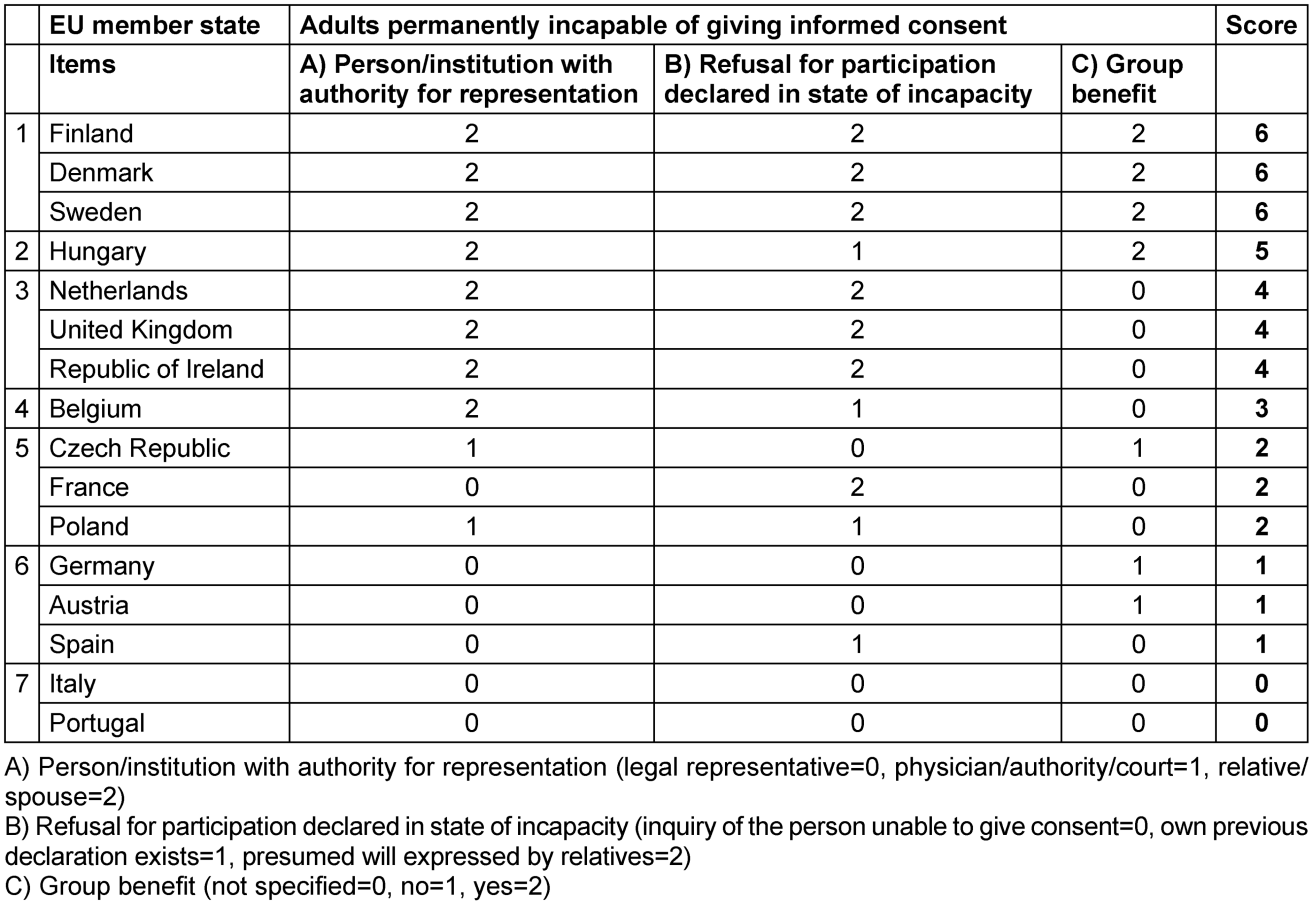
Evaluation of national laws according to selected items for adults permanently incapable of giving informed consent
